# Supplementary material for: Three-dimensional imaging of vascular development in the mouse epididymis
Source: eLife. 2023 Jun 13;12:e82748. doi: 10.7554/eLife.82748 (PMC10264076; doi:10.7554/eLife.82748)
Supplement: Supplementary file 1. [file elife-82748-supp1.docx]

**All data sources in the (*.ims) format can be read with the IMARIS viewer, which can be downloaded free of charge at https://imaris.oxinst.com/imaris-viewer.**

**For Zeiss AxioScan data sources (*.czi) the reading is done with the Zen_lite software downloadable at** [**https://www.zeiss.fr/microscopie/produits/microscope-software/zen-lite.html**](https://www.zeiss.fr/microscopie/produits/microscope-software/zen-lite.html)**.**

**Figure1_data-sources**:

Contains macroscopic images of the lymphatic vasculature of the epididymis and testis in the VEGFR3:YFP model obtained with a Leica binocular loupe and converted to IMARIS format (*.ims). (8 files included: VEGFR3-YFP_Adult.ims; VEGFR3-YFP_Adult_Caput.ims; VEGFR3-YFP_Adult_Corpus.ims; VEGFR3-YFP_Adult_Corpus_Zoom. ims; VEGFR3-YFP_Adult_Cauda.ims; VEGFR3-YFP_Adult_Cauda_2.ims; VEGFR3-YFP_10DPN.ims: VEGFR3-YFP_10DPN_2.ims.

**Figure2_data-sources:**

Contains high resolution three-dimensional (3D) imaging of the blood and lymphatic vasculature of the mouse epididymis after organ clearing in IMARIS format. Two datasets for each part (caput and cauda) of the epididymis are proposed: 1) Figure2_Caput_Blood_3lymph.ims; 2) Figure2_Cauda_Blood_3Lymph. ims which corresponds to the contralateral organ shown in Figure 2B and shows immunostaining with the blood marker PLVAP (red) and 3 lymphatic markers VEGFR3:YFP (green), LYVE1 (cyan) and PDPN (white); 3) Figure2_Caput_4Lymph. ims and 4) Figure2_Cauda_4Lymph.ims with immunostaining for four lymphatic markers (Figure2B) where the VEGFR3:YFP transgene is in green, LYVE1 (cyan), PDPN (white) and PROX1 (magenta).

**Figure3_data-sources:**

Contains two files used as examples for blood vessel (red, left) and lymphatic vessel (green, right) densitometry at the IS (white) and epididymal caput (Figure3_Caput.ims) and for the S10 segment (white) and epididymal caudal (Figure3_cauda.ims). Surface rendering was performed with IMARIS software.

**Figure4_data-sources:**

Contains high resolution three-dimensional (3D) imaging of the blood and lymphatic vasculature of the caput epididymis at different stages of development after organ clearing in IMARIS format. Four files are proposed: Figure4_Caput_10DPN.ims; Figure4_Caput_20DPN.ims; Figure4_Caput_30DPN.ims; Figure4_Caput_40DPN.ims. PLVAP is in red, VEGFR3:YFP in green and LYVE1 in cyan.

**Figure5_data-sources:**

Contains 4 folders named VEGF-A/GAPDH, VEGF-C/GAPDH, VEGF-D/GAPDH, VEGFR3/GAPDH. Each contains a pdf with the source_blots with annotations and the ChemiDoc (Biorad) image sources of the western blot presented in the publication plus a second replicate. One for the VEGF ligand and the other for the corresponding GAPDH blot. The VEGFR3/GAPDH file contains in addition to the GAPDH blot, the complete and truncated blot of VEGFR3 presented in the publication. We had to cut the membrane because the signal given by the cross-reactivity of the secondary antibody (anti-Rat) on the heavy and light chains of immunoglobulins disturbed the detection of the VEGFR3 receptor band. In the literature, the molecular weight of the monomeric receptor varies between 150 and 120 kDa depending on the source. It is the band at 120 kDa that we measured for quantification.

The covariance data sources presented in figure 5 C are presented in a file named Figure5_covariance.xlsx

**Figure6_data-sources:**

Contains all the immunostaining data sources shown in Figure 6 in IMARIS (.ims) or Zen (.czi) format. Figure6_A_VEGF-A_Organ_Scan.czi; Figure6_A_VEGFR2; Figure6_VEGF-A_Caput.ims; Figure6_VEGF-A_Corpus.ims; Figure6_VEGF-A_Cauda.ims; Figure6_B1_VEGF-C_VEGF-D_Organ_Scan.czi; Figure6_B2_VEGF-C_VEGF-D. ims; Figure6_B3_VEGF-C_VEGF-D.ims; Figure6_B4_VEGF-C_VEGF-D.ims; Figure6_B5_VEGF-C_VEGF-D.ims; Figure6_B6_VEGF-C_VEGF-D.ims; Figure6_B7_VEGF-C_VEGF-D.ims; Figure6_B8_VEGF-C_VEGF-D.ims; Figure6_B9_Control.ims.

**Figure7_datasources:**

Contains 1 acquisition file (Figure 7A.ims) after a 3D CROP was performed on the region of interest at the caput septa and 4 very high magnification acquisition files (20X0.95 Leica objective) of the S8-S9/S10 septa region at different stages of postnatal development (Figure7B_10DPN.ims; Figure7B_20DPN.ims; Figure7B_30DPN.ims; Figure7B_40DPN.ims and Figure7B_Adult.ims). Surface renderings were made with the IMARIS software module. PLVAP is in red, VEGFR3:YFP is in green, LYVE1 is in cyan PROX1 is in magenta and PDPN is in white.

**Figure10_datasources:**

**Contain the xlsx file of the table presented in figure 10B**

**Figure2_figSup1_datasources:**

Includes control image files obtained by confocal microscopy and converted to IMARIS format (Figure2-figSuppl1A and Figure2-figSuppl1B) and a high-resolution light sheet imaging file (Figure2-figSuppl1C).

**Figure2_figSup2_datasources:**

Contains one file (Figure2-figSuppl2_epididymis.ims) of High-resolution three-dimensional (3D) imaging of the Blood (PLVAP in red) and lymphatic (VEGFR3:YFP in green) vasculatures of the complete mouse epididymis organ clearing in IMARIS format.

**Figure2_figSup3_datasources:**

Contains high-resolution three-dimensional (3D) imaging of the blood and lymphatic vasculature of the mouse epididymis after organ clearing in IMARIS format. Two data sets for each part (caput and cauda) of the epididymis are proposed: 1) Figure2_Caput_Blood_3lymph.ims and 2) Figure2_Cauda_Blood_3Lymph. ims correspond to the contralateral organ shown in Figure 2B and show immunostaining for the blood marker PLVAP (red) and 3 lymphatic markers VEGFR3:YFP (green), LYVE1 (cyan), PDPN (white); 3) Figure2_Caput_4Lymph. ims and 4) Figure2_Cauda_4Lymph.ims with immunostaining for four lymphatic markers (Figure2B) where the VEGFR3:YFP transgene is in green, LYVE1 (cyan), PDPN (white) and PROX1 (magenta). The file Figure 2_figSuppl3B presents the surface rendering of a crop view of the initial segment of the caput of the adult epididymis. The PLVAP (red), the VEGFR3:YFP transgene (green) and the PDPN (white) are present in the data source but in the publication only PLVAP and VEGFR3:YFP are present. The magnification is 12X.

**Figure4_figSup1_datasources:**

Contains high resolution three-dimensional (3D) imaging of the blood and lymphatic vasculature of the cauda epididymis at different stages of development after organ clearing in IMARIS format. Four files are proposed: Figure 4_figSuppl1_Cauda_10DPN.ims; Figure 4_figSuppl 1_20DPN.ims; Figure 4_figSuppl1_Cauda 30DPN.ims; Figure 4_ figSuppl1_Cauda _40DPN.ims. PLVAP is in red, VEGFR3:YFP is in green and LYVE1 is in cyan.

**Figure2_Video_datasources:**

Includes 3 files that allowed the realization of the videos presented in this work corresponding to the caput (Figure2_Video1.ims), corpus (Figure2-Video2.ims) and cauda (Figure2-Video3.ims) regions of the epididymis.
